# Association between 5q23.2-located polymorphism of *CTXN3* gene (Cortexin 3) and schizophrenia in European-Caucasian males; implications for the aetiology of schizophrenia

**DOI:** 10.1186/s12993-015-0057-9

**Published:** 2015-03-17

**Authors:** Omar Šerý, Jan Lochman, Jana Povová, Vladimír Janout, Jiří Plesník, Vladimir J Balcar

**Affiliations:** Laboratory of Neurobiology and Molecular Psychiatry, Laboratory of Molecular Physiology, Department of Biochemistry, Faculty of Science, Masaryk University, Kotlářská 2, 611 37, Brno, Czech Republic; Institute of Animal Physiology and Genetics, Academy of Sciences, Veveří 97, 602 00, Brno, Czech Republic; Department of Epidemiology and Public Health, Faculty of Medicine, University of Ostrava, Ostrava, Czech Republic; Laboratory of Neurochemistry, Bosch Institute and Discipline of Anatomy and Histology, School of Medical Sciences, Sydney Medical School, The University of Sydney, 2006 Sydney, NSW AUSTRALIA

**Keywords:** DISC1, SLC12A2, NKCC1, GABAergic neurotransmission, Amyloid precursor protein (APP), Alzheimer’s disease

## Abstract

**Background:**

The objective of the study was to examine several polymorphisms in *DISC1* and *CTNX3* genes as possible risk factors in schizophrenia. *DISC1* (disrupted-in-schizophrenia 1) has been studied extensively in relation to mental disease while *CTXN3*, has only recently emerged as a potential “candidate” gene in schizophrenia. *CTXN3* resides in a genomic region (5q21-34) known to be associated with schizophrenia and encodes a protein cortexin 3 which is highly enriched in brain.

**Methods:**

We used ethnically homogeneous samples of 175 male patients and 184 male control subjects. All patients were interviewed by two similarly qualified psychiatrists. Controls were interviewed by one of the authors (O.S.). Genotyping was performed, following amplification by polymerase chain reaction (PCR), using fragment analysis in a standard commercial setting (Applied Biosystems, USA).

**Results:**

We have found a statistically significant association between rs6595788 polymorphism of *CTXN3* gene and the risk of schizophrenia; the presence of AG genotype increased the risk 1.5-fold. Polymorphisms in *DISC1* gene showed only marginally statistically significant association with schizophrenia (rs17817356) or no association whatsoever (rs821597 and rs980989) while two polymorphisms (rs9661837 and rs3737597) were found to be only slightly polymorphic in the samples.

**Conclusion:**

Evidence available in the literature suggests that altered expression of cortexin 3, either alone, or in parallel with changes in DISC1, could subtly perturb GABAergic neurotransmission and/or metabolism of amyloid precursor protein (APP) in developing brain, thus potentially exposing the affected individual to an increased risk of schizophrenia later in life.

## Background

There are numerous reports in the literature, including those on genome-wide association studies (GWAS), proposing putative links between particular genes and mental diseases such as schizophrenia. *DISC1* (Disrupted-in-Schizophrenia 1) is one such “candidate” gene (reviews: [1,2]) and this is so despite extensive investigations having produced, to date, little evidence for a direct association between any structural changes in *DISC1* and a specific disease (review: [3]). However, the protein which *DISC1* encodes (DISC1) is known to be involved in the development of the central nervous system; neural proliferation and migration as well as neurite outgrowth are among the most often cited targets of DISC1 [2,4].

In contrast to *DISC1*, *CTXN3,* a three-exon (exons 1a, 1b, 2 and 3) gene spread over a 9.6-kb region of human chromosome 5q23.2, has been known and studied for only a few years [5-8]. In humans, *CTXN3* translates into a protein (cortexin 3 also known as KABE: “Kidney And Brain Expressed” protein) which is present mainly in kidney and brain, including foetal brain tissue [5].

Recently, Panichareon *et al.* [6] described an association between two *CTXN3* polymorphisms and schizophrenia in a sample of Thai Asian population. This finding is intriguing for a variety of reasons. Panichareon *et al.* [6] noted a linkage disequilibrium (albeit a moderate one) between SNP’s in *CTXN3* and *SLC12A2*; both these genes are within the 5q23 region that has been identified as a “locus of vulnerability” or a “candidate region” with respect to genetic risk of schizophrenia [9-11]. Furthermore, genetic studies have associated a gene-interplay between *SLC12A2* and *DISC1* with an altered risk of schizophrenia [12]. In fact, *SLC12A2* has been linked to *DISC1*, also as a result of *in vitro* experiments [12] and following *in vivo* measurements of hippocampal activity in humans [13]. By analogy with the putative role of *DISC1*, the above observations can be taken as implying that altered genetics of *CTXN3,* either individually or in conjunction with changes in *DISC1*, might represent a significant risk factor in schizophrenia. This conjecture forms the basis for our current hypothesis.

As we have previously carried out several successful case-control association studies between schizophrenia and SNP’s in *OPRM1, DRD3, SNAP-25, MTHFR* and *ADRA2A* genes in samples of typical European population [14-16] we decided to use a similar approach to test the present hypothesis and include both *CTXN3* and *DISC1* gene polymorphisms in our investigation. We now report on the rs6595788 polymorphism of *CTXN3* gene and the rs17817356, rs821597, rs9661837, rs980989 and rs3737597 polymorphisms of *DISC1* gene and discuss them as possible risk factors for pathophysiology of schizophrenia in a group of male patients and controls. All these polymorphisms have been studied by other authors in associations studies related to psychiatric diseases [17-21].

## Methods

### Subjects

In order to eliminate from our data possible confounding influence of sex-differences in the aetiology of schizophrenia [22], we used all-male samples of both patients and controls. The total of 359 males of Czech nationality (Caucasians) entered the study. The group of patients with schizophrenia included 175 males (mean age 35.5 ± 10.9) hospitalized at the Department of Psychiatry, Faculty Hospital, Brno and the Psychiatric Hospital, Jihlava. The patients were diagnosed according to ICD-10 criteria (International Classification of Diseases, 10th Edition) and according to DSM-IV criteria (APA, 1994). All patients underwent structured interviews with qualified psychiatrists (cf. Acknowledgments). Patients with psychiatric comorbidities were excluded from the study.

The control group included 184 males (mean age 48.2 ± 13.8). They were volunteers recruited from blood donors at Blood Bank Brno, patients treated for erectile dysfunction at Trauma Hospital Brno, among employees of private companies in Brno, agriculture farms in the area around Brno, university academics and employees of National Theatre in Brno. The Mini-International Neuropsychiatric Interview (M.I.N.I.) was performed with each member of the control group [23]. Any individuals suspected of not being fully mentally healthy were excluded from the group. In order to minimize personal bias, all screening and interviewing was done by only two psychiatrists with similar backgrounds who closely communicated with each other, or, in the case of control subject, by one of the authors (O.S.) assisted by a qualified psychologist.

All participants, whether they entered as patients or controls, signed an informed consent to participate in the study. Genotypes of the participants were analysed only after the interviews with psychiatrists had been completed. The project was approved by the Ethical Committee of the Faculty Hospital, Brno.

### Genotyping

DNA was extracted from 200 mL of EDTA-anticoagulated whole blood using an UltraClean Blood DNA Isolation Kit (Mobio, USA). Six SNPs (rs6595788, rs17817356, rs821597, rs9661837, rs980989 and rs3737597) were assayed using multiplexed polymerase chain reaction (PCR) amplification, followed by single base extension (SNaPshot, Applied Biosystems, USA). The primers used for multiplexed PCR and the SNaPshot method are listed in Table 1. Each multiplex polymerase chain reaction was done in a final volume of 20 mL. The reaction mixture consisted of 2 mL of DNA template (50 ng/mL), 0.2 mM primers (Table 1) and 2 x Kappa 2G FAST Ready Mix (Kappa Biosystems). After initial denaturation at 95°C for 3 min, samples were amplified through 40 cycles (95°C for 10 sec, 60°C for 20 sec with 50% ramp, 72°C for 50 sec), followed by holding at 72°C for 10 min in a Veriti thermal cycler (Applied Biosystems, USA). After purification with PCR ExoSAP (Fermentas, Lithuania), PCR products were mixed and 1 mL of mixture was added to SNaPshot reaction mix (Applied Biosystems, USA) in a total volume of 10 mL. Cycling conditions were set up according to the manufacturer’s instruction manual. After the SNaPshot reaction, SAP (Fermentas, Lithuania) treatment was carried out for 30 min at 37°C. One microliter of each sample was then added to 9 mL of deionized formamide and 0.4 mL of standard size LIZ 120 (Applied Biosystems, USA) before analysis on a 3100 DNA Fragment Analysis System (Applied Biosystems, USA) in a 36 cm capillary array using the POP-7 polymer.

**Table 1:** **Sequences of the primers used for genotyping by Multiplex PCR and SNaPshot analysis**

**SNP**	**Primers**	**Multiplex**	**Primer for SNaPshot extension**
rs6595788	GATGGGCTGTTTTGACACCTA	A	(T) _12_TGGTTTAGGAATATTTAAAATACAGACAGC
	CAACGCCAAATGATGGTAGAT		
rs17817356	GGAGAATGTGAGTCATGATGTGA	A	(T)_4_ATAACTAAACGTTCAAGTATTTCCCT
	ATCTGACCAAAATCAGGCACA		
rs821597	GGTCCAGAGACATGAGTTTGC	A	(T)_9_GAGTTTGCCATCAGGCAATAATGAATT
	ATGGCCAAACCTTGCTTTAGT		
rs9661837	ATGAACTGGTCACATGGCACT	B	(T)_4_TGGCACTTGGAATCCTTGAGTT
	CACTTGGGCTAGTGACGGTTA		
rs980989	CCAGAAACATGTAACGGTTGG	B	TGCCATGCTAAGCCCTTTACA
	ATGCTGCCTGTCTCTGACTGT		
rs3737597	CAAATGGCACAGGAAAAAGAG	B	(T)_10_CTATTCTCAAATCCTGTGGAAGACATTC
	CAGTGGAAAGGTGGTTCATGT		

### Statistical analysis

The CSS Statistica software (StatSoft, USA) was used for statistical evaluation of the results. The chi-square test was used for the comparison of genotype frequencies in the studied groups. Odds ratios (OR’s) and 95% confidence intervals (95% CI) as estimates of relative risk for the schizophrenia associated with the genotypes were calculated using logistic regression. To minimize false-positive results potentially caused by multiple testing, we applied the Bonferroni correction for three independent loci genotyped. The level of statistical significance was adjusted to P = 0.008. Hardy-Weinberg equilibrium was tested by chi^2^ test.

## Results

Allele and genotype frequencies of all analysed polymorphisms are displayed in Table 2. Preliminary statistical evaluation indicated no genetic linkages among the studied *DISC1* gene polymorphisms (data not shown). We found a statistically significant association between schizophrenia and rs6595788 polymorphism of *CTXN3* gene. The frequency of G allele is significantly higher in schizophrenic patients in comparison with control subjects (p = 0.0018). Genotype frequencies are significantly different between patients and controls (p = 0.004). The presence of G allele in the genotype increased the risk of schizophrenia (Odds Ratio = 1.6923; 95% CI of OR = 1.2231 - 2.3414, Risk Ratio = 1.1674; 95 % CI of OR = 1.0602 - 1.2855).

**Table 2:** **Genotype frequencies of CTXN3 and DISC1 gene polymorphisms among cases and controls**

**Genes**	**SNP**	**Genotype**	**Controls (** ***N*** **= 184)**		**Patiens (** ***N*** **= 175)**		**OR**	**(95% CI)**	***P*** **value**
			**Number**	**(%)**	**Number**	**(%)**			
*CTXN3*	rs6595788	AA	106	57.6	70	40.2	1.00	-	-
		AG	67	36.4	86	49.4	1.94	(1.52-3.02)	**0.003**
		GG	11	6.0	18	10.4	2.48	(1.10-5.65)	0.025
		AG+GG	78	42.4	104	59.8	2.02	(1.33-3.08)	**0.001**
*DISC1*	rs17817356	GG	74	40.2	50	28.9	1.00	-	-
		AG	80	43.5	99	57.2	1.83	(1.15-2.91)	0.010
		AA	30	16.3	24	13.9	1.18	(0.62-2.26)	0.609
		AG+AA	110	59.8	123	71.1	1.65	(1.06-2.57)	0.025
*DISC1*	rs821597	GG	74	40.2	70	40.2	1.00	-	-
		AG	89	48.4	78	44.8	0.93	(0.59-1.45)	0.737
		AA	21	11.4	26	15.0	1.31	(0.68-2.54)	0.424
		AG+AA	110	59.8	104	59.8	1.00	(0.66-1.53)	0.998
*DISC1*	rs980989	CC	123	66.9	108	62.1	1.00	-	-
		AC	53	28.8	59	33.9	1.27	(0.81-1.99)	0.303
		AA	8	4.3	7	4.0	1.00	(0.35-2.84)	0.995
		AC+AA	61	33.1	66	37.9	1.23	(1.80-1.90)	0.345
*DISC1*	rs9661837	AA	183	99.5	169	96.6	NC*		
		AG	1	0.5	6	3.4			
		GG	0	0.0	0	0.0			
		AG+GG	1	0.5	6	3.4			
*DISC1*	rs3737597	CC	171	92.9	166	95.4	NC**		
		CT	13	7.1	7	4.0			
		TT	0	0	1	0.6			
		CT + TT	13	7.1	8	4.6			

All chi-squared tests are two-tailed. Alpha value is adjusted by Bonferroni correction and statistically significant results (P<0.008) are marked bold.

NC – minor allele frequency to low to calculate precise OR and P values.

*the statistical significance of difference in allele frequencies by Fisher’s exact test is p = 0.06.

**the statistical significance of difference in allele frequencies by Fisher’s exact test is p = 0.4.

Only a marginal statistically significant difference between patients and controls was noted in rs17817356 polymorphism of *DISC1* gene, with AG genotype apparently more frequent in patients (p = 0.01). When analysing rs9661837 polymorphism, we found no GG genotype in either schizophrenics or controls. AG genotype was present in 6 schizophrenic patients and in only one control subject and the statistical significance of difference in allele frequencies (p = 0.06) and in genotype frequencies (p = 0.048) could be considered as marginal at best. For rs9661837 and rs3737597 polymorphisms we did not perform OR and RR analyses because of too low minor allele frequencies. We detected no relationship between schizophrenia and rs821597, rs980989 and rs3737597 polymorphisms of the *DISC1* gene.

Genotypic frequency of rs17817356 polymorphism (*DISC1*) in schizophrenic patients but not in the controls deviated from Hardy-Weinberg equilibrium (p < 0.03). The interaction between rs6595788 and rs17817356 polymorphisms appeared significant, too (p < 0.002); the genotype AGAG having been found to be about 2x more frequent in schizophrenic patients than in control subjects (Table 3). This genotype alone could, therefore, contribute to a higher risk of schizophrenia.

**Table 3:** **Frequencies of the most important haplotypes in patients with Schizophrenia versus Control Subjects**

**Genes**	**SNPs**	***p-value*** **(chi-squared test)**	**SNPs alleles**	**Estimated frequencies**	
				**Case**	**Control**
***CTXN3*** * ***DISC1***	rs6595788 * rs17817356	0.002	AG*AG	0.283	0.125
			AA*GG	0.116	0.201
			GG*AG	0.075	0.022

## Discussion

There have been two previous attempts to establish an association between polymorphisms of *CTXN3* gene and schizophrenia. One was performed in the United States using brain *in vivo* imaging as a quantitative trait (QT) enhancement of statistical power in a genome-wide association study (QT-GWAS; [7]), the other one was a case-control association analysis of a group of Thai Asians [6]. The present study is, therefore, the first one of its kind carried out entirely within Europe on a sample of typical European-Caucasian population [24]. It is also, to date, the largest case-control study, in terms of the number of subjects surveyed, of association between a *CTXN3* polymorphism and any mental disease.

Cortexin 3 belongs to “cortexin family” that includes three proteins: cortexin 1, cortexin 2 and cortexin 3. Cortexin 3 displays amino acid sequences very similar (about 43% homology in humans) to those found in a protein cortexin 1 (encoded by *CTXN1* gene), previously identified in the cerebral cortex [25]. Human *CTXN1* gene is located on the chromosome 19p13.2 and it has 2 exons, *CTXN2* with 5 exons is located in 15q21.1 chromosome region (according to recent information from GenBank). Two alternative variants of *CTXN3* cDNA sequences differ in the 5′ untranslated region implying a possibility of alternative splicing regulating tissue-specific expression of the gene. Indeed, *in silico* cloning has indicated that brain and kidney each express own forms of cortexin 3 which differ from one another, particularly in the region encoded by exon 1 [5]. Additional theoretical considerations [5] indicated that cortexin 3 could be an integral membrane protein involved in extracellular or intracellular signalling.

Using expressed sequence tag (EST) analyses of cDNA libraries, orthologs of cortexin 3 with highly conserved sequences have been identified in mouse, rat, cow, dog, zebrafish, chicken, chimpanzee, Rhesus monkey and frog ([5]; cf. GenBank). Non-human forms of cortexin 3 have species-characteristic tissue distributions but seem to be always enriched in brain. Cortexins 1-3 should not be confused with another “cortexin” (“(r)-cortexin”), a 43 kDa nitric oxide synthase activating protein from kidney, studied mostly in the context of blood pressure regulation and related disorders [26,27]. “Cortexin” may promote growth of neurites [28] and it has been claimed that it can restore cognition after ischemic stroke [29].

While we found little or no association between the status of the subjects and polymorphisms in *DISC1*, in the case of *CTXN3*, there was a clear, statistically significant, albeit quantitatively modest, link between rs6595788 and schizophrenia. This observation could have implications for the aetiology of schizophrenia, at least for the male form(s) of the disease. What would be the most likely responsible mechanism(s)?

*CTXN3* - in analogy to *DISC1* - has been shown to interact with *SLC12A2* [6,8,10,12,13]. *CTXN3* and *SLC12A2* genes are located at chromosome 5, in the region that is highlighted as the second most important region linked to the schizophrenia in a meta-analytical study by Lewis et al. [10]. It is also within the region linked to neurocognitive traits associated with higher risk of schizophrenia as reported by Almasy et al. [30]. According to our *in silico* analysis, both polymorphism described by Panichareon [6] as associated with the schizophrenia (rs 698172 and rs245178) are located in an intergenic region adjacent to *CTXN3* gene that has recently been shown to contain a sequence corresponding to a non-coding RNA of unknown function. The rs6595788 polymorphism, which is a subject of our study, is located directly at 5′-end of *CTXN3* gene, its precise locus being at a distance of 16787 bp from the proximate polymorphism studied by Panichareon [6] (rs698172) found in the intergenic region at the opposite side of the *CTXN3* sequence i.e. downstream of 3′-end of the gene.

*SLC12A2* encodes a transporter which co-transports Cl^**-**^, Na^+^ and K^+^ (NKCC1 a.k.a. SLC12A2; solute carrier 12A2). NKCC1 carries Cl^**-**^ into the cells and is functionally closely linked to the ionotropic GABA receptors (iGABAR’s) which function as Cl^**-**^-permeable ligand- (GABA-) gated channels. However, the relationship between iGABAR’s and NKCC1 exists mainly in the developing brain tissue, resulting in GABA acting on iGABAR’s as a neuron-depolarizing (i.e. “excitatory”) signalling molecule. In fact, it has been known for some time that, in the developing rat cortex, GABA is released in a controlled, stimulus-coupled, Ca^2+^-dependent fashion well before the formation of GABAergic synapses [31], probably as a regulator of neuronal network development [32]. Transition to the adult function of GABA as an inhibitory neurotransmitter occurs when NKCC1 is supplanted by KCC2 (SLC12A5), a transporter that carries Cl^**-**^ out of the cells thus allowing iGABAR’s to become hyperpolarizing (review: [33]). It has been suggested that perturbations in the timing of the transition from NKCC1 to KCC2 and the ensuing switch from depolarization to hyperpolarisation could cause subtle structural and functional changes in brain tissue eventually leading to mental disease [34] (review: [33]). This could constitute the mechanism for the polymorphisms in the *SLC12A2*-associated genes *DISC1* and *CTNX3* to influence the developmental process and contribute to the aetiology of schizophrenia. Moreover, there is a male *v*. female difference in the developmental timing of the depolarization/hyperpolarization switch [35]. If this difference translates into sex-specific effects on the brain development (and a sex-specific effect on the risk of schizophrenia), our choice of all male population increased homogeneity of the sample and could have significantly improved the statistical power of the present study.

Presence of another potentially relevant mechanism involving cortexin 3 has been indicated by a recent report by Chouraki et al. [36]. They performed a genome-wide association meta-analysis on more than three thousand healthy subjects studying plasma levels of amyloid beta (Aβ) peptides. They established that the plasma levels of Aβ _1-42_ were most closely associated with the rs11241936 polymorphism of *CTXN3* gene. Subsequent *in vitro* studies showed that cortexin 3 interfered with amyloid precursor protein (APP) metabolism and decreased the secretion of Aβ-fragments. Involvement of APP and Aβ fragments in Alzheimer's disease is well known (reviews: [37-39]) but their role in the normal brain, particularly during the development, has been given less attention [40]. In fact, APP is present in growth cones and it has a role in the formation of neurites and synaptogenesis [41]. Mice lacking APP displayed dramatically altered brain morphology [42] possibly as a result of disrupted migration of neural precursor cells in the developing cortex [43]. DISC1 has also been studied in the context of neuronal development and migration and shown to interact with APP [4] thus providing another biochemical locus where cortexin 3 and DISC1 could act together.

APP has also been shown to influence the expression of NR1, a protein subunit of critical importance in the formation of functional NMDA receptors [44]. Changes in cortexin 3/DISC1/APP interactions could, therefore, result in altered expression and distribution of NMDA receptors as has been observed in schizophrenia [45] (review: [46]). Any such relationships are, however, likely to be extremely complex and involve additional genetic (or epigenetic) mechanisms [45]; their detection may depend on the development and application of new analytical technologies (review: [47]).

In conclusion, we report a strong association between schizophrenia and a single nucleotide polymorphism in the *CTXN3* gene (cortexin 3) in an ethnically homogenous group of male patients. In contrast, we found only weak or no associations between schizophrenia and several polymorphisms in *DISC1* gene. Available evidence suggests that cortexin 3 is involved in brain ontogeny, particularly in the development of GABAergic neurotransmission and metabolism of APP which could, in turn, impact on neuronal maturation, migration and synaptogenesis. Taking into account the developmental hypothesis of schizophrenia, we conjecture that any genetic variations in the *CTXN3* gene affecting expression and/or characteristic of cortexin 3 protein would have a potential to contribute to the aetiology of complex mental diseases.
